# The E3 ubiquitin ligase TRIM25 regulates adipocyte differentiation via proteasome-mediated degradation of PPARγ

**DOI:** 10.1038/s12276-018-0162-6

**Published:** 2018-10-15

**Authors:** Jae Min Lee, Sun Sil Choi, Yo Han Lee, Keon Woo Khim, Sora Yoon, Byung-gyu Kim, Dougu Nam, Pann-Ghill Suh, Kyungjae Myung, Jang Hyun Choi

**Affiliations:** 1 Department of Biological Sciences, Ulsan National Institute of Science and Technology (UNIST), Ulsan, 689-798 Korea; 2 Center for Genomic Integrity (CGI), Institute for Basic Science (IBS), Department of Biological Sciences, Ulsan National Institute of Science and Technology (UNIST), Ulsan, 689-798 Korea

## Abstract

Peroxisome proliferator-activated receptor gamma (PPARγ) is a ligand-dependent transcription factor that regulates adipocyte differentiation and glucose homeostasis. The transcriptional activity of PPARγ is regulated not only by ligands but also by post-translational modifications (PTMs). In this study, we demonstrate that a novel E3 ligase of PPARγ, tripartite motif-containing 25 (TRIM25), directly induced the ubiquitination of PPARγ, leading to its proteasome-dependent degradation. During adipocyte differentiation, both TRIM25 mRNA and protein expression significantly decreased and negatively correlated with the expression of PPARγ. The stable expression of TRIM25 reduced PPARγ protein levels and suppressed adipocyte differentiation in 3T3-L1 cells. In contrast, the specific knockdown of TRIM25 increased PPARγ protein levels and stimulated adipocyte differentiation. Furthermore, TRIM25-knockout mouse embryonic fibroblasts (MEFs) exhibited an increased adipocyte differentiation capability compared with wild-type MEFs. Taken together, these data indicate that TRIM25 is a novel E3 ubiquitin ligase of PPARγ and that TRIM25 is a novel target for PPARγ-associated metabolic diseases.

## Metabolic disorders: TRIM-ming the fat

A protein that targets peroxisome proliferator-activated receptor γ (PPARγ) (a key regulator of fat cell formation) for degradation suppresses the formation of fat. Excess fat can lead to obesity and cause type 2 diabetes and cardiovascular disease, yet little is known about the mechanisms through which fat cells arise from progenitor cells. Jang Hyun Choi and colleagues at the Ulsan National Institute of Science and Technology, Korea, have found that TRIM25, a protein implicated in cancer and antiviral immune responses, reduces the activity of PPARγ by adding a small regulatory protein that acts as a signal for degradation. Reducing the levels of TRIM25 in progenitor cells increases their ability to differentiate into fat cells. These findings suggest that TRIM25 could be a useful target for developing new therapies against obesity and metabolic disorders.

## Introduction

Adipose tissue plays a pivotal role in storing excess energy and is a center for energy metabolism^1^. Excess body fat is considered one of the major causes of insulin resistance, dyslipidemia, type 2 diabetes, certain types of cancer, and cardiovascular disease^2^. With respect to obesity, adipocytes exhibit an altered energy homeostasis status to not only store energy but also to generate and secrete hormones and cytokines called adipokines^1,3^. For instance, the expression of insulin resistance-inducing adipokines, including tumor necrosis factor-α, interleukin-1, and resistin is increased in the adipose tissue of obese individuals, whereas the production of the insulin-sensitizing hormones adiponectin or adipsin is decreased^4^. Furthermore, defects in adipocyte differentiation or function increase the probability of metabolic disorders. Thus, understanding the detailed molecular mechanisms of adipose tissue biology, especially adipocyte differentiation, may provide insights for the treatment of obesity and metabolic syndromes.

Adipocyte differentiation is tightly controlled by a series of transcription factors. A number of studies have demonstrated that peroxisome proliferator-activated receptor gamma (PPARγ) and CCAAT/enhancer binding proteins (C/EBPs) are the primary regulators of adipogenesis and have been shown to have broad overlap in their transcriptional targets^5,6^. Interestingly, C/EBP-α-null embryonic fibroblast cells fail to undergo adipogenesis, but this defect can be restored by the overexpression of PPARγ^7,8^. Conversely, forced expression of C/EBP-α in PPARγ-null embryonic fibroblast cells prevents the cells from differentiating^7^. These experiments demonstrated that PPARγ is the key transcriptional regulator of adipogenesis and is both sufficient and necessary for fat cell differentiation.

The transcriptional activity of PPARγ is well known to be upregulated by its ligands, such as thiazolidinediones (TZDs)^9^. PPARγ specifically heterodimerizes with retinoid X receptor (RXR) and binds DNA repeats of the sequence AGGTCA (DR1 elements), and the PPARγ/RXR heterodimer regulates a variety of target genes in different cells^10^. In the resting state (in the absence of PPARγ ligands), PPARγ preferentially binds to nuclear receptor corepressor 1 (NCOR1) and silencing mediator for retinoid and thyroid receptor (SMRT/NCOR2)^11^. These complexes recruit chromatin-modifying enzymes such as histone deacetylases to repress transcription^12,13^. However, corepressors become dissociated from PPARγ when it is activated by ligands. Then, coactivators, such as steroid receptor coactivators, PPARγ coactivator 1s, histone acyltransferases, and the mediator complexes, are recruited and interact with PPARγ to promote gene transcription^10,14,15^. In addition to ligands, post-translational modifications (PTMs), including phosphorylation, SUMOylation, acetylation, and ubiquitination, are considered as some of the major processes regulating the transcriptional activity of PPARγ^16,17^. Phosphorylation of PPARγ at Ser112 by mitogen-activated protein kinase suppresses PPARγ transcriptional activity and adipocyte differentiation^18^. In contrast, while PPARγ phosphorylation at Ser273 by cyclin-dependent kinase 5/ERK does not change its transcriptional activity, this modification has important implications for the treatment of type 2 diabetes^19,20^. SUMOylation of PPARγ at Lys107 blocks its transcriptional activity, while SUMOylation at Lys395 represses inflammatory gene expression by blocking nuclear factor kappa B (NF-κB) activation^13,21,22^. PPARγ is also ubiquitinated and degraded in a proteasome-dependent manner^23,24^. Together, these studies have shown that PTMs of PPARγ are important factors in the physiological roles of PPARγ. Thus, characterization of the novel PTMs of PPARγ will provide important insights into our understanding of the physiological function of PPARγ with respect to adipogenesis.

Tripartite motif (TRIM) proteins are defined as E3 ubiquitin ligases, as they contain a ring-finger domain^25,26^. To date, more than 77 TRIM proteins have been identified in humans that are involved in a broad range of biological processes. TRIM25 (also known as EFP) is a downstream target of estrogen receptor α^27^. The expression of TRIM25 is upregulated in response to estrogen and is believed to mediate the effects of estrogen with respect to breast cancer as a primary response gene^28^. In addition, TRIM25 induces the ubiquitination of retinoic acid-inducible gene 1 and regulates host antiviral innate immunity^29^. In this study, we report a novel role of TRIM25 in regulating metabolic pathways by mediating PPARγ ubiquitination and its proteasome-dependent degradation, suggesting that TRIM25 may be a potential therapeutic target in PPARγ-mediated metabolic diseases such as obesity and type 2 diabetes.

## Materials and methods

### Cell culture

3T3-L1, HCT116, human embryonic kidney (HEK)-293T, and HEK-293 cells were obtained from American Type Culture Collection (Manassas, VA) and cultured in Dulbecco’s modified Eagle’s medium (DMEM) containing fetal bovine serum (Gemini Bio-Products, West Sacramento, CA) and 1% penicillin/streptomycin (Thermo Fisher Scientific, Waltham, MA) at 37 °C under a humidified 5% CO_2_ atmosphere. Adipocyte differentiation was induced by treating cells with DMEM medium containing 10% fetal bovine serum, 0.5 mM isobutylmethylxanthine, 1 μM dexamethasone, and 850 nM insulin. Two days after the induction, the inducing medium was replaced with maintenance DMEM medium containing 10% FBS and 850 nM insulin. Lipid accumulation in the cells was detected by Oil Red O staining. Wild-type (WT) mouse embryonic fibroblast cells (MEFs) and TRIM25-knockout (KO) MEFs were kindly provided by Dr. Kyung-soo Inn (College of Pharmacy, Kyung Hee University, Korea)^29^. All chemicals used for cell culture were obtained from MilliporeSigma (St. Louis, MO).

### Plasmid constructs and RNA interference

The mammalian expression vector for FLAG-epitope tagged WT PPARγ was described previously^19^. WT human TRIM25 and its deletion mutants were kindly provided by Dr. V. Narry Kim^30^. siRNAs for TRIM25 #1 (5′-CCTCGACAAGGAAGATAAA-3′) and #2 (5′-GCATCTGCTACGGAAGCAT-3′) were purchased from Shanghai GenePharma (Shanghai, China). HCT116 cells were transfected with the siRNAs using Lipofectamine RNAi MAC (Thermo Fisher Scientific, Waltham, MA). The sequences used for lentiviral short hairpin RNA (shRNA) expression vectors (pLKO.1; Dharmacon, Lafayette, CO) targeting TRIM25 were #1 (5′-TTCCTCAGTTTGTACTCCAGG-3′) and #2 (5′-ATGATCCAGATCTATCTTAGG-3′). For lentiviral production, HEK-293T cells were transfected with 10 µg of the lentiviral vectors. Following infection of the cells with the viral vectors, 3T3-L1 cells were selected by incubation with 2 μg/ml puromycin (MilliporeSigma, St. Louis, MO).

### Binding assay, immunoprecipitation, and antibodies

Glutathione *S*-transferase (GST)-fused proteins (PPARγ domain mutants) immobilized with glutathione-agarose were incubated with TRIM25-expressing cell lysates for 2 h at 4 °C. Protein complexes were pulled down by centrifugation and washed four times with binding buffer. Precipitates were detected by immunoblotting using anti-GST or TRIM25 antibodies. For analyzing interactions between endogenous PPARγ and TRIM25, 3T3-L1 adipocytes were lysed with binding buffer. Cell lysates were incubated with anti-PPARγ or TRIM25 antibodies and analyzed by western blotting. HEK-293 cells expressing PPARγ, TRIM25, or their mutants were lysed in binding buffer, and total cell lysates were incubated with an anti-hemagglutinin (HA) antibody at 4 °C. Immunoprecipitants or total cell lysates were analyzed with specific antibodies as indicated. The antibodies used in this study included α-TRIM25, α-PPARγ, α-GST, α-Ub, α-aP2, and α-adipsin antibodies, which were purchased from Santa Cruz Biotechnology (Dallas, TX), while α-HA, α-actin, α-HSP90, and α-adiponectin were purchased from Cell Signaling Technology (Danvers, MA).

### Identification of PPARγ-binding complexes

PPARγ-null MEFs^7^ were cultured in DMEM containing 10% fetal bovine serum. Nuclear extracts were prepared from PPARγ-null MEFs that stably expressed FLAG-PPARγ^WT^ and were incubated with the immobilized FLAG M2 agarose gel, which was then washed with binding buffer and incubated with FLAG peptide to elute the bound proteins. Immunoprecipitated proteins were separated by SDS-polyacrylamide gel electrophoresis, and specific bands were excised and digested with trypsin. The digested proteins were then analyzed by reverse-phase liquid chromatography combined with tandem mass spectrometry (LC-MS/MS) using a high-resolution hybrid mass spectrometer (LTQ-Orbitrap; Thermo Scientific, Waltham, MA). All MS/MS spectra were searched against the Uniprot protein sequence database using SequestHT (Thermo Scientific, Waltham, MA).

### Reporter gene assay

HEK-293 cells were transfected with the pDR-1 luciferase reporter plasmid, PPARγ, RXRα, and pRL-Renilla using Lipofectamine 2000 (Thermo Fisher Scientific, Waltham, MA). Following an overnight transfection, the cells were treated with rosiglitazone for 24 h. Subsequently, the cells were harvested and used to perform reporter gene assays using a Dual-Luciferase kit (Promega, Madison, WI). Luciferase activity was normalized to Renilla activity.

### Gene expression analysis

Total RNA was isolated from cells or tissues using TRIzol reagent (Thermo Fisher Scientific, Waltham, MA). RNA was reverse-transcribed using an ABI Reverse Transcription Kit. Quantitative PCR reactions were performed with SYBR green fluorescent dye on an ABI9300 PCR machine (Supplementary Table 1). Relative mRNA expression was determined using the ΔΔ−Ct method and was normalized to tata-binding protein levels.

### In vitro ubiquitination assay

FLAG-TRIM25 was transiently expressed in HEK-293 cells, purified using anti-FLAG M2 affinity gel, and eluted by adding 3 × FLAG peptide according to the manufacturer’s instructions. Recombinant GST-PPARγ protein was incubated with 200 ng E1 (UBE1, Boston Biochem, Cambridge, MA), 500 ng E2 (UbcH5c, Boston Biochem, Cambridge, MA), 10 μg ubiquitin, and 2 mM ATP in the absence and presence of TRIM25^WT^ or TRIM25^CS^ in 60 μl of reaction buffer (40 mM Tris-HCl, pH 7.6, 50 mM NaCl, and 1 mM dithiothreitol) for 1 h at 37 °C. After incubating, reactions were pulled down with glutathione-agarose and washed four times with binding buffer, followed by western blotting using α-Ub or α-PPARγ antibodies. Reaction mixtures were directly assayed by western blotting using an α-TRIM25 antibody to assess TRIM25 self-ubiquitination as a control.

### Correlation analysis in human adipose tissues

TPM-normalized RNA-sequencing data from 338 subcutaneous adipose tissue samples were obtained from the GTEx portal using the sample labels downloaded from ArrayExpress^31^. Next, the Pearson correlation coefficient between TRIM25 and PPARγ within the fat samples was calculated after removing one outlier sample whose expression in either gene was more than 5 standard deviations from the mean. The two genes showed a significant negative correlation of −0.248 (*p*-value 4.14E-6).

### Animals

All animal experiments were performed according to procedures approved by the Ulsan National Institute of Science and Technology’s Institutional Animal Care and Use Committee. Five-week-old male C57BL/6J mice (DBL, Chungbuk, Korea) were fed a high-fat diet (60% kcal fat, D12492, Research Diets Inc., New Brunswick, NJ) for 10 weeks. The *ob/ob* mice used in this study were purchased from the Jackson Laboratory (Bar Harbor, ME).

## Results

### TRIM25 interacts with PPARγ

**Fig. 1:**
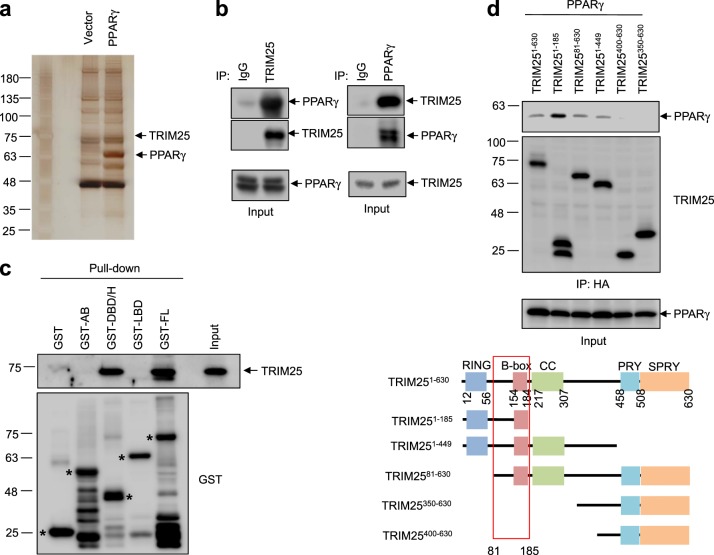
TRIM25 interacts with PPARγ. **a** Silver staining of PPARγ and its associated proteins. FLAG-tagged PPARγ was purified using FLAG M2 agarose. **b** The PPARγ complexes were immunopurified from adipocytes, and endogenous TRIM25 was detected by western blotting. **c** GST-fused fragments of PPARγ (A/B, LBD, DBD/H, and FL) were incubated with cell lysates containing TRIM25. The GST-fragment complexes were analyzed by western blotting. **d** HA-tagged deletion mutants of TRIM25 were generated and expressed with PPARγ in HEK-293 cells. From the HA-TRIM25 complexes, PPARγ was analyzed by western blotting

To identify potential PTM modulators of PPARγ, we performed proteomic analyses of binding complexes formed with PPARγ. As shown in Fig. 1a, several bands of potential interest were observed and subjected to LC-MS/MS. Among the multiple PPARγ-associated proteins identified, TRIM25 was of particular interest because it functions as a ubiquitin E3 ligase and as an ISG15 E3 ligase^29,32^. TRIM25 has also been reported to mediate polyubiquitination of the DDX58 N-terminal CARD-like region, which is crucial for triggering the cytosolic signal transduction that leads to the production of interferons in response to viral infection^33^. These data led us to investigate whether TRIM25 plays a physiological role in regulating both the PTMs and the transcriptional activity of PPARγ.

To confirm the interaction between TRIM25 and PPARγ, PPARγ was immunoprecipitated from cultured adipocytes and TRIM25 was detected by immunoblotting. As shown in Fig. 1b, PPARγ was observed to interact with endogenous TRIM25. Structural aspects of the interaction between TRIM25 and PPARγ were further investigated in vitro using recombinant PPARγ fragments, including the A/B region (the transcriptional regulatory region), the DNA-binding domain/hinge region (DBD/H) and the ligand-binding domain. As shown in Fig. 1c, TRIM25 specifically interacted with the DBD/H domain of PPARγ. To further identify the specific region required for TRIM25 binding to PPARγ, smaller regions of TRIM25 were expressed into HEK-293 cells and coimmunoprecipitated with TRIM25. As shown in Fig. 1d, amino acids 81–185 of TRIM25 were necessary for its interaction with PPARγ.

### The E3 ligase activity of TRIM25 is required to decrease PPARγ protein stability

**Fig. 2:**
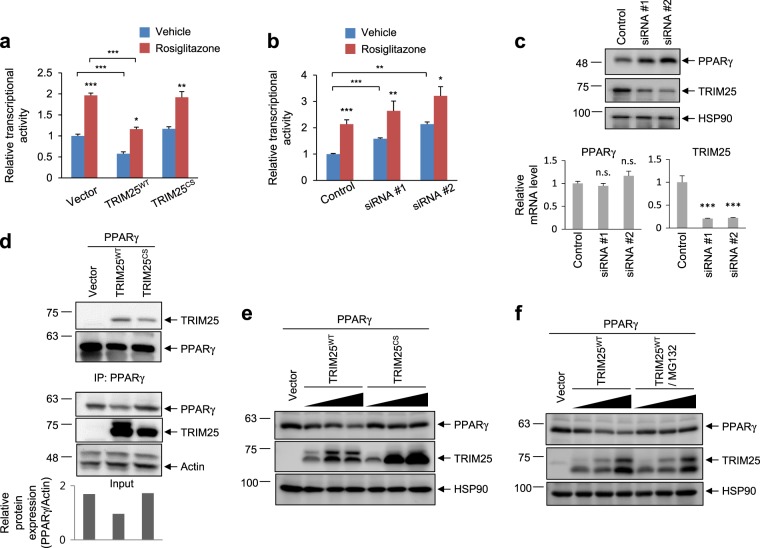
TRIM25 degrades PPARγ through the proteasomal pathway. **a**, **b** TRIM25 represses the transcriptional activity of PPARγ. HEK-293 cells were transfected with TRIM25^WT^ and TRIM25^CS^ with PPARγ (**a**) or siRNAs against TRIM25 (#1 and #2) with PPARγ (**b**) with or without rosiglitazone. Cells were analyzed with a luminometer (*n* = 3). **c** The TRIM25 protein level was increased by TRIM25 siRNAs in HCT116 cells. The PPARγ and TRIM25 protein levels were analyzed by western blotting, and their mRNA levels were analyzed by real-time quantitative PCR (RT-qPCR) (*n* = 3). **d** HEK-293 cells were transfected with PPARγ, TRIM25^WT^, or TRIM25^CS^. The interaction between TRIM25 and PPARγ was analyzed by western blotting. Relative PPARγ protein levels were measured using ImageJ (bottom graphs). **e** HEK-293 cells were transfected with TRIM25^WT^ or TRIM25^CS^ with PPARγ in dose-dependent manner. Indicated proteins were measured by western blotting. **f** HEK-293 cells were transfected with TRIM25^WT^ with PPARγ in dose-dependent manner with or without MG132 (10 μM for 6 h). The indicated proteins were measured by western blotting. All error bars shown are the s.e.m. (*p < 0.05, **p < 0.01, ***p < 0.001)

To elucidate the role of TRIM25 as an E3 ligase, we generated an E3 ligase-defective TRIM25 mutant by mutating cysteines 50 and 53 to serine (C50S/C53S, TRIM25^CS^), which does not possess E3 ubiquitin ligase activity^34^. First, we investigated the effect of TRIM25 on PPARγ transcriptional activity. A luciferase assay was performed in HEK-293 cells expressing a peroxisome proliferator response element-containing luciferase construct. As shown in Fig. 2a, the overexpression of TRIM25^WT^ decreased PPARγ transcriptional activity. Importantly, TRIM25^CS^ did not suppress PPARγ transcriptional activity, indicating that the WT E3 ubiquitin ligase is important for regulating PPARγ transcriptional activity. Consistent with this result, specific knockdown of TRIM25 using two different small interfering RNAs (siRNA #1 and #2) increased PPARγ transcriptional activity (Fig. 2b). Furthermore, similar effects were observed when PPARγ was treated with rosiglitazone, a synthetic PPARγ ligand. Two different TRIM25 siRNAs increased PPARγ protein levels without altering mRNA levels (Fig. 2c). Together, these results suggest that TRIM25 regulates PPARγ protein levels in a manner that is dependent on its E3 ligase activity.

After establishing that TRIM25 is an E3 ligase that regulates PPARγ protein levels, we next investigated the effect of TRIM25 on PPARγ protein stability. While both TRIM25^WT^ and TRIM25^CS^ interacted with PPARγ, TRIM25^WT^ induced PPARγ degradation, whereas TRIM25^CS^ did not (Fig. 2d). Furthermore, an increase in TRIM25^WT^ expression resulted in decreased PPARγ protein expression in a dose-dependent manner, which was not observed for TRIM25^CS^ (Fig. 2e), indicating that the E3 ligase activity of TRIM25 is involved in PPARγ degradation. The overexpression of TRIM25 significantly reduced PPARγ protein levels, which was restored by treating cells with MG132, a specific proteasome inhibitor (Fig. 2f). Together, these results strongly suggest that proteasomal degradation of PPARγ is mediated by TRIM25.

**Fig. 3:**
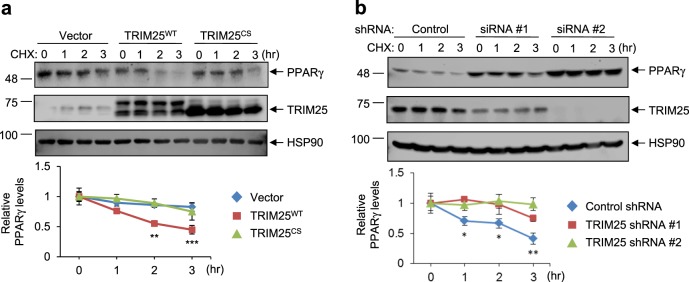
TRIM25 destabilizes PPARγ. HCT116 cells were transfected with TRIM25^WT^ and TRIM25^CS^ (**a**) or siRNAs against TRIM25 (#1 and #2) (**b**) in the presence or absence of CHX (50 μg/ml for the indicated time). The indicated proteins were measured by western blotting. Relative PPARγ protein levels were measured using ImageJ (bottom graphs)

Next, we used a cycloheximide chase assay to measure the half-life of PPARγ in cells that overexpressed or were downregulated for TRIM25. As shown in Fig. 3a, compared with control cells, the half-life of PPARγ was significantly reduced by the overexpression of TRIM25^WT^ but not TRIM25^CS^. In addition, the specific knockdown of TRIM25 could stabilize PPARγ, suggesting that TRIM25 is a key regulator of PPARγ protein stability (Fig. 3b).

### TRIM25 is an E3 ligase that mediates the ubiquitination of PPARγ

**Fig. 4:**
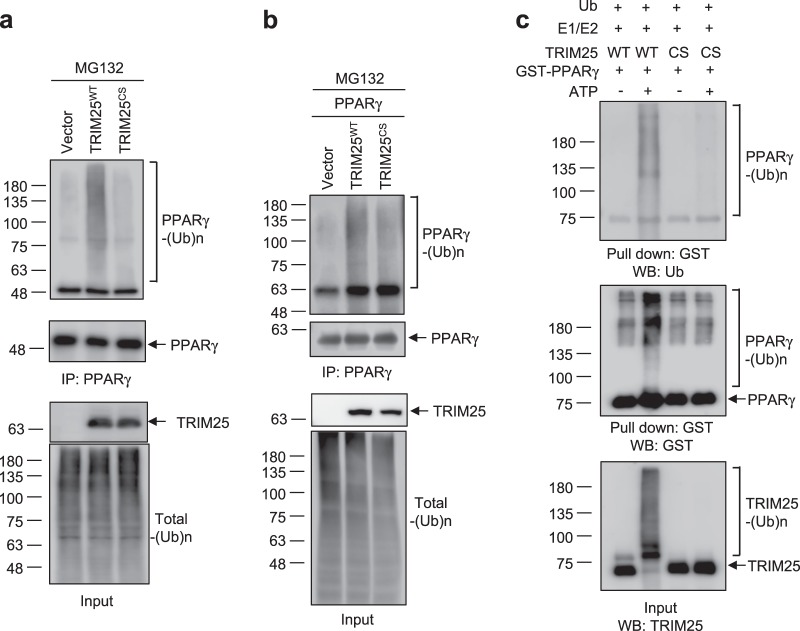
TRIM25 mediates the ubiquitination of PPARγ through its E3 ligase activity. **a** 3T3-L1 cells were stably expressed with TRIM25^WT^ or TRIM25^CS^ and differentiated for 6 days. Cells were treated with MG132 for 6 h. Cell lysates were incubated with an α-PPARγ antibody, and ubiquitination of PPARγ was analyzed by western blotting. **b** HEK-293 cells were transfected with TRIM25^WT^ or TRIM25^CS^. Cell lysates were incubated with an α-FLAG antibody, and ubiquitination of PPARγ was analyzed by western blotting. **c** Ubiquitination of PPARγ by TRIM25 in vitro. Purified GST-tagged PPARγ was incubated with E1, E2, ubiquitin (Ub), TRIM25^WT^, or TRIM25^CS^ in the absence and presence of ATP as indicated. Ubiquitination of PPARγ was analyzed by western blotting using the indicated antibodies

Next, we performed a ubiquitination analysis to further assess whether TRIM25 functions as a specific E3 ligase of PPARγ. We used a retroviral system to generate 3T3-L1 cell lines that stably overexpressed TRIM25^WT^ or TRIM25^CS^ or carried empty vector (control cell line), and the cells were fully differentiated into adipocytes. Endogenous PPARγ from cultured adipocytes was immunoprecipitated to assess the ubiquitination of PPARγ by western blotting (Fig. 4a). Although the PPARγ protein levels were decreased in cells expressing TRIM25^WT^, TRIM25^WT^ expression resulted in increased PPARγ ubiquitination when cells were pretreated with MG132, which was not observed following TRIM25^CS^ expression. Consistent with these results, transiently expressed TRIM25^WT^ enhanced PPARγ ubiquitination compared with the TRIM25^CS^ mutant in HEK-293 cells (Fig. 4b). To further confirm the direct effect of TRIM25 on the ubiquitination of PPARγ, we performed an in vitro ubiquitination assay using purified TRIM25 and PPARγ. As expected, TRIM25 induced the ubiquitination of recombinant PPARγ, while TRIM25^CS^ did not (Fig. 4c). These results suggest that PPARγ is a substrate of TRIM25.

### TRIM25 is negatively correlated with PPARγ

**Fig. 5:**
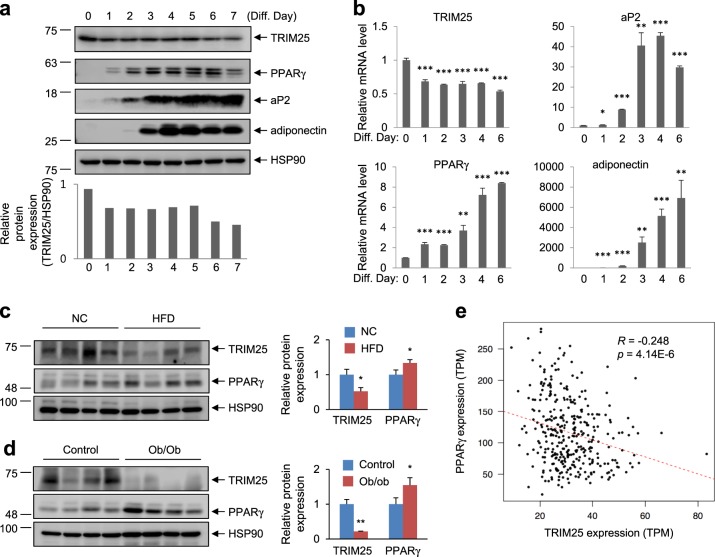
Negative correlation between TRIM25 and PPARγ expression both in mice and humans. **a** Immunoblot analysis of TRIM25, PPARγ, and adipogenic markers during 3T3-L1 cell differentiation. Relative TRIM25 protein levels were measured using ImageJ (bottom graphs). **b** Real-time quantitative PCR analysis of the mRNA expression of TRIM25, PPARγ, and adipogenic markers during 3T3-L1 cell differentiation. **c** Immunoblot analysis of TRIM25 and PPARγ in white adipose tissue (WAT) of normal chow-fed (NC) vs. high-fat-fed (HFD) mice. **d** Immunoblot analysis of TRIM25 and PPARγ in WAT of control vs. *ob/ob* mice. Relative TRIM25 and PPARγ protein levels were measured using ImageJ (right graphs). **e** Pearson correlation coefficients between TRIM25 and PPARγ levels within human subcutaneous fat samples were calculated. All error bars shown are the s.e.m. (*p < 0.05, **p < 0.01, ***p < 0.001)

In the present study, our results demonstrated that TRIM25 regulates PPARγ protein stability. Thus, we next investigated the physiological role of TRIM25 in adipocyte differentiation. First, we examined the physiological relevance of TRIM25 and PPARγ during adipocyte differentiation. Western blot and real-time PCR analyses revealed that TRIM25 was expressed in pre-adipocytes and that its expression was significantly decreased during adipogenesis (Fig. 5a, b). In contrast with TRIM25 expression, the expression of PPARγ and adipogenic marker genes, including aP2 and adiponectin, were significantly increased, indicating that TRIM25 expression is negatively correlated with that of PPARγ. Next, we measured TRIM25 expression in adipose tissue from high-fat-diet (HFD)-induced obesity model mice and genetically induced obesity model mice (*ob/ob*). As shown in Fig. 5c, d, TRIM25 expression was significantly downregulated in adipose tissue from both HFD and *ob/ob* mice compared with control mice, whereas PPARγ expression was dramatically upregulated. To examine whether the correlation between TRIM25 and PPARγ expression in adipose tissue is relevant in humans, we used RNA-sequencing data of 338 human adipose tissue samples from the Genotype-Tissue Expression (GTEx) project to analyze the correlation between TRIM25 and PPARγ gene expression^35,36^. As shown in Fig. 5e, the expression of TRIM25 was significantly negative correlated with that of PPARγ. Taken together, these results suggest that the expression of TRIM25 is inversely correlated with that of PPARγ in both mice and humans.

### TRIM25 suppresses adipocyte differentiation

**Fig. 6:**
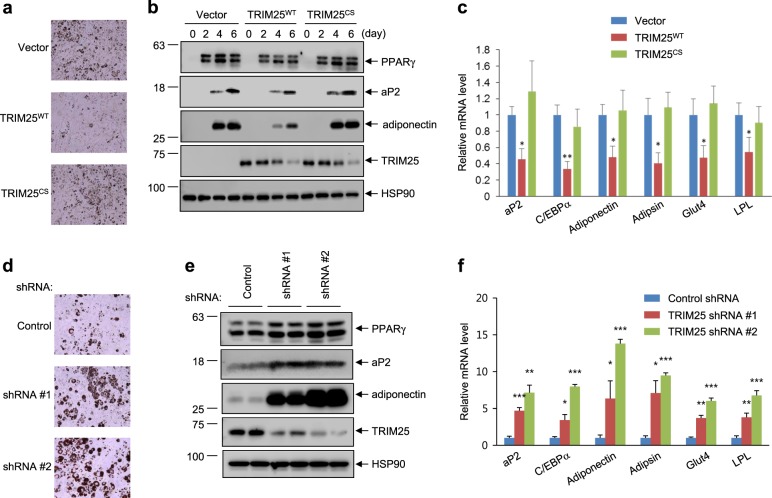
TRIM25 suppresses adipocyte differentiation in 3T3-L1 cells. Stably expression of TRIM25^WT^ and TRIM25^CS^ (**a**–**c**) or shRNAs (#1 and #2) for TRIM25 (**d**–**f**) in 3T3-L1 pre-adipocytes was induced for 6 days. **a**, **d** Differentiated cells were stained by Oil Red O. **b**, **e** The protein expression of TRIM25, PPARγ, and adipogenic markers was analyzed by western blotting. **c**, **f** The mRNA expression of adipogenic markers was analyzed by real-time quantitative PCR. All error bars shown are the s.e.m. (*p < 0.05, **p < 0.01, ***p < 0.001)

PPARγ is both necessary and sufficient for adipogenesis, and alterations in PPARγ activity affect adipogenesis. Consistent with the observed TRIM25-mediated downregulation of PPARγ stability, when TRIM25^WT^ was expressed in differentiating 3T3-L1 cells, triglyceride accumulation was suppressed, as confirmed by Oil Red O staining (Fig. 6a). Furthermore, the protein and mRNA expression levels of adipocyte-selective genes, including aP2, C/EBP-α, adiponectin, adipsin, glucose transporter-4 (GLUT4), and LPL were also reduced (Fig. 6b, c). However, the TRIM25^CS^ mutant did not alter the expression of adipogenic markers. In addition, the specific knockdown of TRIM25 using a lentivirus expressing a shRNA targeting TRIM25 enhanced adipocyte differentiation (Fig. 6d) and the expression of adipocyte-specific proteins and genes compared with that of the control (Fig. 6e, f).

**Fig. 7:**
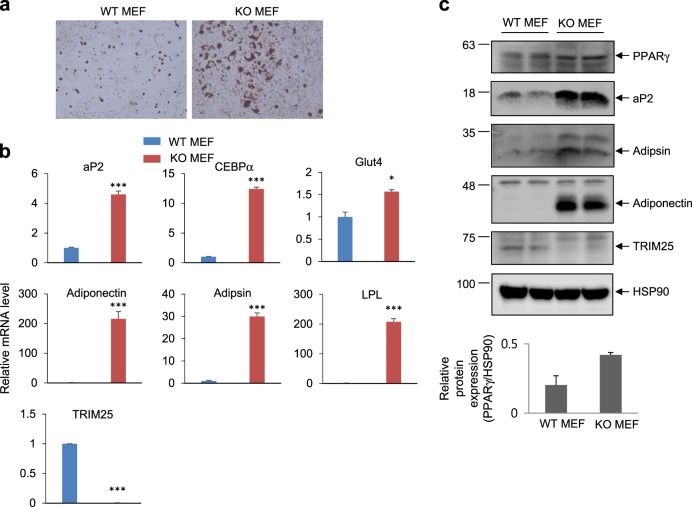
Elimination of TRIM25 induces adipogenesis in mouse embryonic fibroblast (MEF) cells. TRIM25-knockout MEFs were induced for adipocyte differentiation. **a** Differentiated cells were stained with Oil Red O. **b** The mRNA expression of adipogenic markers was analyzed by real-time quantitative PCR. **c** The protein expression of TRIM25, PPARγ, and adipogenic markers was analyzed by western blotting. Relative PPARγ protein levels were measured using ImageJ (bottom graphs). All error bars shown are the s.e.m. (*p < 0.05, ***p < 0.001)

To further confirm the effect of TRIM25 on adipogenesis, we used WT and TRIM25 KO MEFs. Compared with WT MEFs, KO MEFs exhibited dramatically enhanced lipid accumulation (Fig. 7a). Furthermore, the expression of adipogenic markers was significantly increased in KO MEFs compared with WT MEFs (Fig. 7b, c). Taken together, these results suggest that TRIM25 plays a key role in the regulation of PPARγ-dependent adipogenic processes.

## Discussion

Adipose tissue is the center of systemic metabolic regulation in rodents and humans. The dysregulation of adipocyte differentiation and/or physiological function results in metabolic disorders such as type 2 diabetes^37,38^. As PPARγ functions as a master regulator of fat cell differentiation and glucose/lipid metabolism, understanding the mechanisms of PPARγ regulation is crucial for combating metabolic disorders. In this study, we demonstrated that the E3 ubiquitin ligase TRIM25 regulates the protein stability of PPARγ, inhibiting adipogenesis in both 3T3-L1 cells and MEFs. Several lines of evidence support this conclusion. First, TRIM25 directly interacts with PPARγ and increases ubiquitin- and proteasome-mediated PPARγ degradation. Second, specific knockdown of TRIM25 dramatically increases adipocyte differentiation. Finally, the expression of TRIM25 is inversely correlated with that of PPARγ in adipose tissue in both mice and humans.

Although numerous studies have reported on the regulation of PPARγ expression at the transcriptional level, the regulation of PPARγ expression at the protein level has been poorly studied. Recently, multiple lines of evidence have suggested that PPARγ is regulated by PTMs, including phosphorylation, SUMOylation, and ubiquitination^17,24^. In particular, the polyubiquitination of PPARγ is crucial for regulating both its expression and transcriptional activity. MKRN1, an E3 ligase of PPARγ, was observed to induce PPARγ ubiquitination and its proteasome-mediated degradation^39^. However, the ubiquitination of PPARγ does not always increase its degradation. In a report by Kilroy et al.^40^, TZDs increased the expression of E3 ligase, Siah2, and Siah2, which are required for adipogenesis by ubiquitinating PPARγ. In addition, NEDD4, an E3 ubiquitin ligase, interacts with and ubiquitinates PPARγ, which results in increased PPARγ protein stability by inhibiting its proteosomal degradation^41^. In this study, we demonstrated that the ubiquitination of PPARγ by a novel E3 ligase, TRIM25, promoted PPARγ proteosomal degradation and suppressed adipogenesis. These results suggest that there are several distinct pathways for ubiquitin-mediated PPARγ regulation and that each E3 ligase regulates PPARγ stability in response to different cellular signals or conditions in adipocytes.

Recent reports have suggested that the protein stability of PPARγ can be regulated by phosphorylation. Keshet et al.^42^ demonstrated that c-Abl-mediated tyrosine phosphorylation of PPARγ increases its accumulation. In addition, epidermal growth factor receptor has been suggested to induce PPARγ phosphorylation, which could lead to MDM2 E3 ubiquitin ligase-mediated PPARγ ubiquitination and degradation^43,44^. Thus, we tested whether known phosphorylation sites of PPARγ could modulate TRIM25-mediated PPARγ ubiquitination and degradation. TRIM25 reduced the stability of the phospho-defective mutants PPARγ S112A and PPARγ S273A (data not shown)^18,19^. Interestingly, phosphorylation at Y78 of PPARγ dramatically suppressed TRIM25-mediated PPARγ ubiquitination and increased PPARγ accumulation (Supplementary Figure 1). Tyrosine phosphorylation of PPARγ at Y78 is induced by the c-Abl or c-Src kinases, both of which are required for fat cell differentiation^42,45,46^. Therefore, it is likely that the modulation of PPARγ phosphorylation at Y78 is an important regulatory mechanism for its protein stability and fat cell differentiation activity. Further mechanistic studies on the role of Y78 phosphorylation in TRIM25-mediated PPARγ regulation should be conducted in the future.

In this study, we demonstrated that TRIM25 binds to PPARγ in adipocytes and that the expression of TRIM25 and PPARγ is inversely correlated in adipocytes in both mice and humans (Fig. 5). Furthermore, we demonstrated the expression of TRIM25 during adipogenic processes, and both TRIM25 gene and the protein levels were transiently reduced after inducing adipocyte differentiation (Fig. 5). The exact molecular mechanism of how the expression of TRIM25 is reduced during adipogenic differentiation process remains unclear. However, we believe that the decrease in TRIM25-mediated degradation of PPARγ during differentiation, in combination with the increase of PPARγ gene expression and increased activity of PPARγ, directly induces the expression of PPARγ itself or C/EBPα during differentiation. In addition to adipocytes, TRIM25 and PPARγ are also expressed in different cells and tissues, especially tumors^47–49^. Multiple lines of evidence suggest that PPARγ acts as a tumor suppressor because it plays a role in inflammation and glucose metabolism in cancer. The overexpression of PPARγ suppresses cell survival by inhibiting cell proliferation and tumor growth^50^. In contrast, the silencing of PPARγ in cancer has the opposite effect on the activation of the cancer survival pathway^51^. TRIM25 has also been linked to several cancers. In particular, TRIM25 is overexpressed in breast, colorectal, and lung cancers, while PPARγ is downregulated in these cancers compared with normal tissues^52–54^. These results suggest that TRIM25 may act as a crucial regulator of PPARγ in different types of tumors. Although further studies are needed to elucidate the molecular relationship between TRIM25 and PPARγ in tumor progression, our results extend our knowledge regarding the regulation of PPARγ and potential targets for the development of novel therapeutic approaches targeting obesity and metabolic diseases.

## Electronic supplementary material

Supl Table 1

Supplementary Figure 1
