# Supplementary material for: The E3 ubiquitin ligase TRIM25 regulates adipocyte differentiation via proteasome-mediated degradation of PPARγ
Source: Exp Mol Med. 2018 Oct 15;50(10):135. doi: 10.1038/s12276-018-0162-6 (PMC6189217; doi:10.1038/s12276-018-0162-6)
Supplement: Supplementary file 1 — Supl Table 1 [file 12276_2018_162_MOESM1_ESM.doc]

| **QPCR Primer** | **Foward** | **Reverse** |
| --- | --- | --- |
| Mouse TRIM25 | ATGGCTCAGGTAACAAGGGAG | GGGAGCAACAGGGGTTTTCTT |
| Human TRIM25 | GTCTCTACCCAGAACAGTTTCC | ATCCAACACAGGCTGATTCC |
| Mouse PPARγ | GCATGGTGCCTTCGCTGA | TGGCATCTCTGTGTCAACCATG |
| Mouse PPARγ | TCTCTCCGTAATGGAAGACC | GCATTATGAGACATCCCCAC |
| Mouse ap2 | AAGGTGAAGAGCATCATAACCCT | TCACGCCTTTCATAACACATTCC |
| Mouse Adiponectin | TGTTCCTCTTAATCCTGCCCA | CCAACCTGCACAAGTTCCCTT |
| Mouse CEBPα | CAAGAACAGCAACGAGTACCG | GTCACTGGTCAACTCCAGCAC |
| Mouse GLUT4 | GATTCTGCTGCCCTTCTG | ATTGGACGCTCTCTCTCC |
| Mouse LPL | AACAAGGTCAGAGCCAAGAG | CCATCCTCAGTCCCAGAAAAG |

**Supplementary Table. 1. Primer sequences for RT-qPCR**
