# Supplementary material for: The E3 ubiquitin ligase TRIM25 regulates adipocyte differentiation via proteasome-mediated degradation of PPARγ
Source: Exp Mol Med. 2018 Oct 15;50(10):135. doi: 10.1038/s12276-018-0162-6 (PMC6189217; doi:10.1038/s12276-018-0162-6)
Supplement: Supplementary file 2 — Supplementary Figure 1 [file 12276_2018_162_MOESM2_ESM.ppt]

## Slide 1
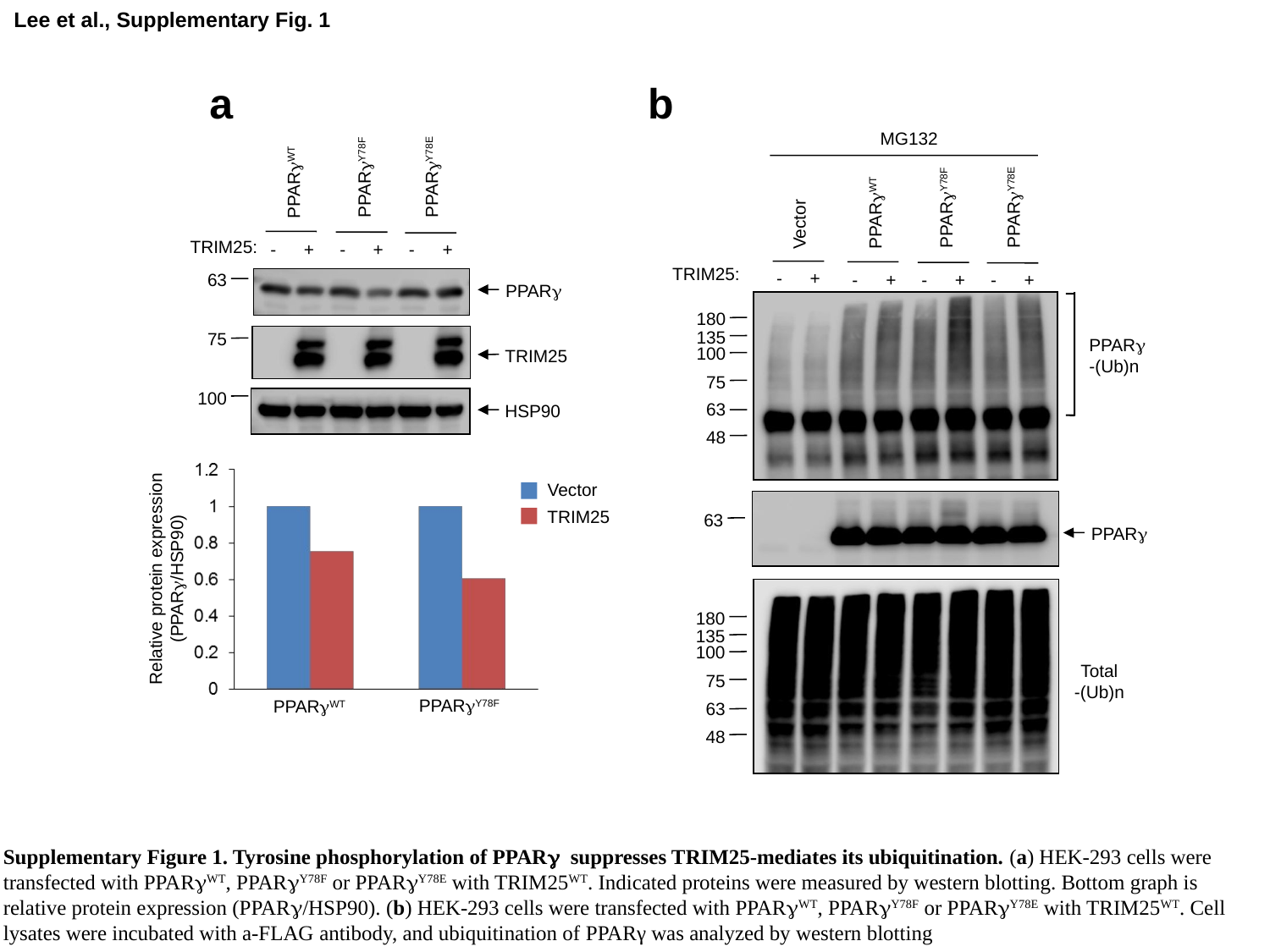

Lee et al., Supplementary Fig. 1
a
b
MG132
PPARγY78E
PPARγY78F
PPARγWT
PPARγY78E
PPARγY78F
PPARγWT
Vector
TRIM25:
-
+
-
+
-
+
TRIM25:
-
+
63
-
+
-
+
-
+
PPARγ
180
135
75
PPARγ
-(Ub)n
100
TRIM25
75
100
63
HSP90
48
Vector
TRIM25
63
PPARγ
Relative protein expression
(PPARγ/HSP90)
180
135
100
Total
-(Ub)n
75
PPARγY78F
PPARγWT
63
48
Supplementary Figure 1. Tyrosine phosphorylation of PPARγ suppresses TRIM25-mediates its ubiquitination. (a) HEK-293 cells were transfected with PPARγWT, PPARγY78F or PPARγY78E with TRIM25WT. Indicated proteins were measured by western blotting. Bottom graph is relative protein expression (PPARγ/HSP90). (b) HEK-293 cells were transfected with PPARγWT, PPARγY78F or PPARγY78E with TRIM25WT. Cell lysates were incubated with a-FLAG antibody, and ubiquitination of PPARγ was analyzed by western blotting
